# Microbial quality and antibiotic sensitivity of bacterial isolates in “Tuo‐Zaafi” vended in the central business district of tamale

**DOI:** 10.1002/fsn3.1216

**Published:** 2019-09-27

**Authors:** Rufina Abas, Samuel J. Cobbina, Godwin Abakari

**Affiliations:** ^1^ Department of Ecotourism and Environmental Management University for Development Studies Nyankpala‐Tamale Ghana

**Keywords:** *E. coli* and antibiotic sensitivity, *Salmonella* spp, *Shigella* spp, *Staphylococcus aureus*, Tamale, Tuo‐Zaafi

## Abstract

**Introduction:**

Food safety concerns remain a challenge across nations and among citizens. Microbial contamination of foods and antibiotic resistance constitutes a global threat to food security. The present study assessed microbial quality of "Tuo‐Zaafi" sold in the Tamale metropolis as well as antibiotic resistance of isolates from these products.

**Results:**

Samples were collected aseptically and transported to the Spanish laboratory complex of the University for Development Studies, Ghana, for microbial analysis. *E. coli* which recorded the highest occurrence was detected in 50% of the T.Z samples with bacterial loads ranging from < 100 to 2.3 × 10^6^ cfu/g (4.49 × 10^5^ ± 5.72 × 10^4^ cfu/g). *Salmonella* spp and *Staphylococcus aureus* recorded the least occurrence representing 33.3%. There were significant differences in the levels of *E. coli, Shigella* spp, *Salmonella* spp, and *Staphylococcus aureus* (*p *< .001) across the four zones demarcated. Results of the antibiotic test revealed higher resistance to the antibiotics employed in the present study (81%). Susceptibility of microbes to ciprofloxacin (100%) was the highest, and higher resistance to gentamycin (100%) was observed in this study.

**Conclusion:**

The study revealed that T.Z sold in the business district of Tamale could constitute a likely health risk to consumers especially when it is consumed in a cold state. It is hereby recommended that the Food and Drugs Authority (FDA) should enforce food hygiene laws and ensure strict adherence.

## 1 INTRODUCTION

Food quality and safety have always been crucial and constitute major aspects of the important factors that promote the successful operation of food trading transactions.

Specifically, at present times food safety issues have placed quality among the very important international food trade concerns. Thus, food quality is viewed as an integrated measure of purity, flavor, texture, color, and appearance that establishes a particular food's acceptability (Giovannucci and Satin, 2000).

Urbanization and changes in consumer habits, including travel, have increased the number of people buying and consuming foods prepared at public places (WHO, 2017). The busy activities and long‐term schedule of individuals per day have also given way to the increasing number of street‐vended foods as well as fast foods. Many of these operations are carried out at locations that do not meet the sanitary qualities and specification stipulated by the food safety bodies (FAO/WHO, 2010). It worth noting that these street foods and fast foods to a large extent contribute significantly toward reducing food insecurity and hunger. That notwithstanding, major concerns have been raised with regard to the hygienic status of these foods during production and marketing (Ogidi, Victor, & Bamidele, 2016). In developed countries, food protection from microbial hazards has received special considerations and attention from stakeholders in food safety institutions and among the public. However, but for several developing countries, the much‐needed consideration and attention has not been given to food safety issues. Worse still, several foodborne disease outbreaks are being underreported in most developing countries especially African countries of which Ghana is not exempted (Adetunji, Hezekiah, & Charity and Tajudeen, 2014).

The annual deaths of an estimated two million people including children are linked to consumption of unsafe food (WHO, 2015). A condition can quickly change or evolve into a more serious state over time that needs varying amounts of resources, coordination, and management to control it. It has also been reported that food safety events that need intervention to safeguard the health of consumers will range from minor incidents to major crises (WHO, 2010).

The past decades have been characterized with rising concerns about antibiotic resistance which is directly associated with food quality and safety (Al‐Waili, Al‐Ghamdi, Ansari, Al‐Attal, & Salom, 2012; CDC, 2000). Antibiotic resistance is noticed when bacteria are able to resist the effect of antibiotics or medicines used to control them. This often results in expensive medical costs, extended hospitalization, and high rates of mortality. Thus, the incidence of antibiotic resistance in the food industry is staged as one of the greatest challenges to global health and food security (WHO, 2018). New resistance mechanisms keep evolving and spreading globally, putting our ability to treat common infectious diseases a daunting task. The evolving and spreading of resistance is aggravated when antibiotics bought without prescription are used by humans and on animals (WHO, 2017).

Maize otherwise known as corn is scientifically called Zea mays. Globally, maize is one of the most extensively cultivated cereal crops and it is also the staple food of several African communities, especially the traditional societies.

In Africa, especially Ghana, a lot of food varieties are consumed. One of the most cherished foods consumed in the northern part of Ghana is “Tuo‐Zaafi” (T.Z). The main ingredient for T.Z is maize, but millet can be used as well. The millet used can be subcategorized into two: “Nara” the early millet and “Kemolega” guinea corn (Liinaghanas, 2014). Tamale has been tagged as the fastest growing city in West Africa as there is an increase in population which is accompanied by increasing number of food vending establishments (GSS, 2010). The prevailing problem is that all these food vending spots adopt several methods in the processing and preparation of these foods specifically.

“Tuo‐Zaafi” (T.Z) which requires the services of different people during the preparation process to hasten readiness to meet up with the choice or preference of consumers is usually done under unhygienic conditions (Ogidi et al., 2016). According to World Bank/MOFA (2007), one person out of every forty Ghanaians suffers from food‐related diseases yearly.

The incidence of several foodborne diseases is increasing at a fast rate due to the unhygienic methods of preparation and storage of foods. Most microbial causative agents of foodborne diseases have developed resistance to commonly used antibiotics (CSPI, 2013). The resistance of pathogens to many antibiotics encourages pathogen persistence in food processing environments, prolonging treatment of the disease conditions, and the rates of hospitalization and mortality in humans (Adetunji et al., 2014). The insufficient information on the incidence of diseases associated with street‐vended foods in most developing countries does not provide inclusive data on the impacts of street‐vended foods on human health (WHO, 2011). Bacterial resistance to multiple antibiotics became a health problem in the 1980s which is not far from repeating in recent times (Charpentier and Courvalin, 1999). There is currently very little information and scientific analysis on the microbial quality of “Tuo‐Zaafi” which extensively consumed in Tamale. Therefore, the aim of the present study was to assess the microbial quality of "Tuo‐Zaafi" sold in the Tamale metropolis as well as antibiotic resistance of isolates from these products.

## 2 MATERIALS AND METHODS

### 2.1 Study area

The study was conducted in the central business district of Tamale which is described as the heart of the metropolis where diverse and many activities take place.

**Figure 1:**
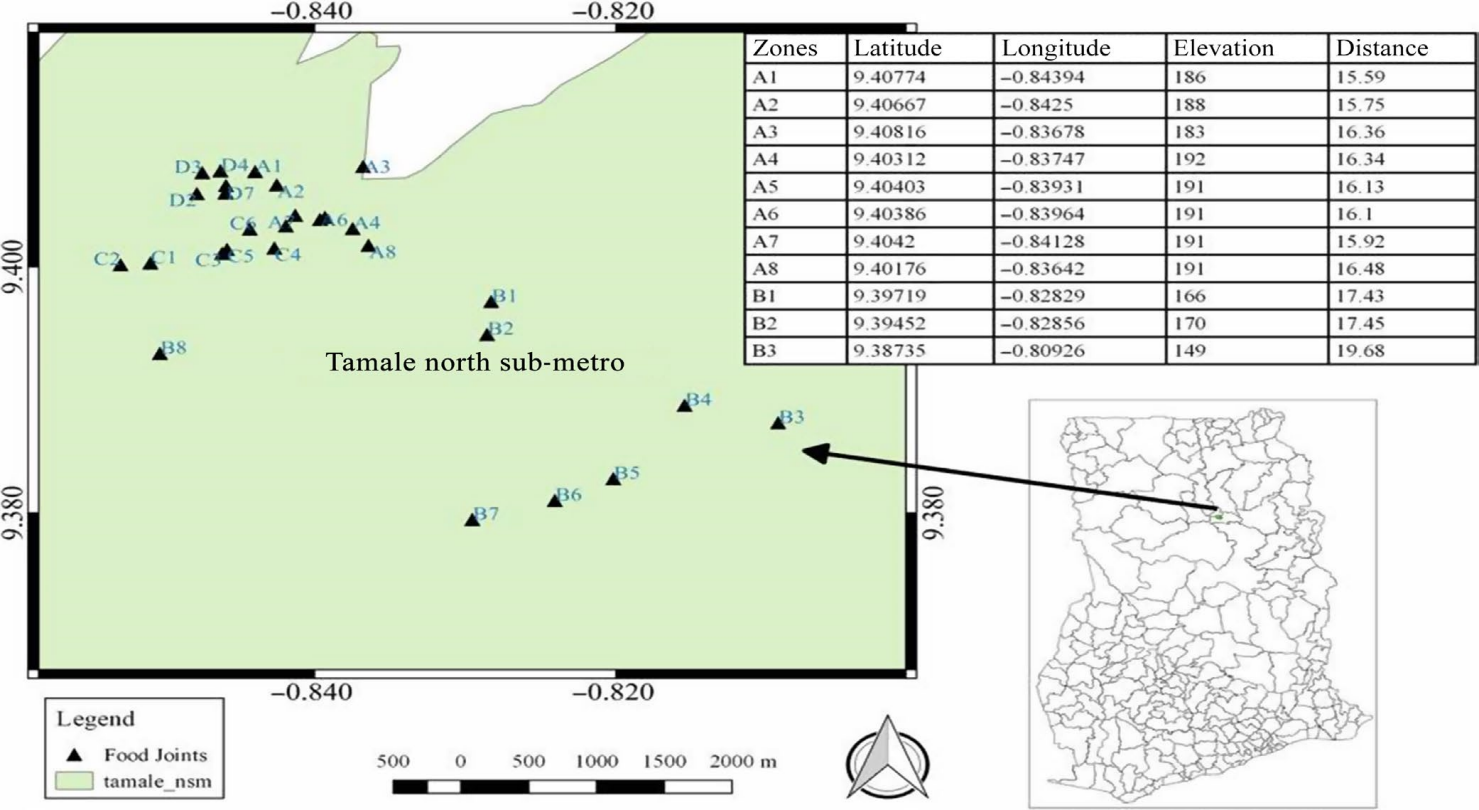
Map of the study area showing the sampled vending sites. ▲; sampling points

Tamale is located in the central part of the northern region and has an estimated land size of 646.90180 km^2^, and it lies between latitude 9°16 and 9°34 north and longitude 0°36 and 0°57 west geographically (GSS, 2010) (Figure 1).

### 2.2 Sample collection and study design

The stratified random sampling procedure was adopted in collecting the T.Z samples. The study area (the central business district of Tamale) was sectioned into four strata (Zones A‐D) based on proximity and similarities of environments of sampling points (Abakari, Cobbina, & Yeleliere, 2018). This was done in order to obtain representative samples from the study area. Zone A included the following communities: Tishigu, Taxi run, Bus top, and Changli. Zone B included communities such as Gumbihini, Moshie Zongo, Parks and gardens, and its environs. Transport yard, Timber market, and Nyohini were categorized under Zone C, and Zone D was made of communities including Zogbeli, Aboabo, and Sabonjida.

A total of thirty (30) “T.Z” samples were purchased from the central business district of Tamale from January to March 2018 using the stratified random sampling technique.

The samples were collected on five different occasions under aseptic conditions. In total, eight (8) samples each were collected from Zones A and C, summing up to a total of 16 samples, and seven (7) samples were collected from Zones B and D summing up to a total of 14 samples. The samples were collected in ziplock polythene sterile bags, kept in ice chest containing ice and transported to the Spanish Laboratory complex of the University for Development Studies, Ghana, for microbial analysis.

Samples were collected within the hours of 10:30 a.m. and 2:00 p.m. as this was the time most of the vendors were out to sell and as well the ripe time for most consumers to patronize the “T.Z.”

### 2.3 Microbial analysis

#### 2.3.1 Sterilization of glassware and equipment

All glasswares including petri dishes were sterilized by autoclaving at 121°C for 15 min. Also, pipette tips together with rubber inoculating loops were sterilized at the same temperature and for the same number of minutes as the petri dishes. Again, the laboratory benches, as well as the laminar flow hood used for the work, were disinfected with 70% isopropyl alcohol.

#### 2.3.2 Preparation of culture media

Media were prepared according to the manufacturer's protocol (Acumedia Manufacturers Inc). The culture media that were used during the study included nutrient agar, plate count agar, xylose lysine deoxycholate agar, Levine eosin‐methylene blue agar, and mannitol salt agar. Mueller‐Hinton agar (Oxoid, UK) was used for the antibiotic test.

#### 2.3.3 Sample preparation for microbial analysis

Twenty‐five grams (25 g) of each T.Z sample was measured into sterile bags under a laminar flow hood. The T.Z in each germ‐free bag was mixed with 225 ml of peptone water. This mixture was homogenized very well by simple “hand massaging” and constantly joggled to obtain a consistent solution (stock solution). Ten (10)‐fold serial dilutions were carried out at five levels.

0.1 ml each of 100 (stock), 10^1^, 10^2^, 10^3^, 10^4^, and 10^5^ dilutions was taken aseptically under the laminar flow hood and inoculated on the appropriate media. The inoculated plates were inverted and incubated at 37°C for 24 hr (APHA, 2008).

#### 2.3.4 Bacterial identification and counting

The bacterial colonies were identified based on colony color. A greenish colony depicted the presence of *Escherichia coli* on the LEMB agar, while colorless or whitish colonies without black centers depicted the presence and growth of *Shigella* spp on the XLD agar and reddish‐pink colonies with black centers were depicted as *Salmonella* spp on the XLD agar and yellowish colonies on the MSA agar showed the presence of *Staphylococcus aureus*. The number of bacterial colonies was established using the American Public Health Association (APHA) (2008) method.

#### 2.3.5 Antibiotic sensitivity test

The disk diffusion agar method was used to determine the antimicrobial sensitivity of the identified isolates. An agar plate with solidified nutrient agar was spread with 0.1 ml of sample of the stock solution with a sterile inoculating loop. Pure colonies were then transferred from the nutrient agar onto Muller‐Hinton agar (Oxoid, UK) plates by streaking for antibiotic test. This procedure was conducted carefully under aseptic conditions with the aid of the laminar flow hood. This was followed by placement of the antibiotic disks on media plates on which the bacterial isolates were inoculated. In total, fifteen different antibiotic disks were used. All procedures were conducted under aseptic conditions under the laminar flow hood.

The plates were incubated for 24 hr at 37°C. The inhibition zones of the various antibiotic disks were observed and measured with the aid of a meter rule, and the results were recorded accordingly.

### 2.4 Data analysis

The ANOVA (analysis of variance) was used to check for significant differences in terms of microbial loads using Genstat (12th edition) software. Microsoft Excel software was used for finding means, standard deviation, and drawing graphs.

## 3 RESULTS

### 3.1 Mean counts of microbes in the T.Z samples

The results of microbial counts from the present study are presented in tables and graphs in order to establish the presence and levels of bacteria of concern identified. The empirical data below present the dynamics of bacterial occurrence identified in this study.

Generally, higher mean counts (cfu/g) of bacteria were observed in Zone B in the T.Z samples collected for the purpose of this study. Notably, Zone A recorded the next highest level of mean counts of bacteria, and this was followed by Zone C with Zone D recording the least mean counts of bacteria (Table 1).

**Table 1:** Results of bacterial isolates with their mean (±SEM) values

Bacteria	Minimum (cfu/ml)	Maximum (cfu/ml)	Mean (±SEM) cfu/ml
*Escherichia coli*	<100	2.3 × 10^6^	4.49 × 10^5^ ± 5.72 × 10^4^
*Shigella* spp	<100	7.3 × 10^6^	5.1 × 10^5^ ± 1.36 × 10^5^
*Salmonella* spp	<100	1.2 × 10^6^	2.65 × 10^5^ ± 4.12 × 10^4^
*Staphylococcus aureus*	<100	1.25 × 10^6^	3.73 × 10^4^ ± 2.14 × 10^4^

**Figure 2:**
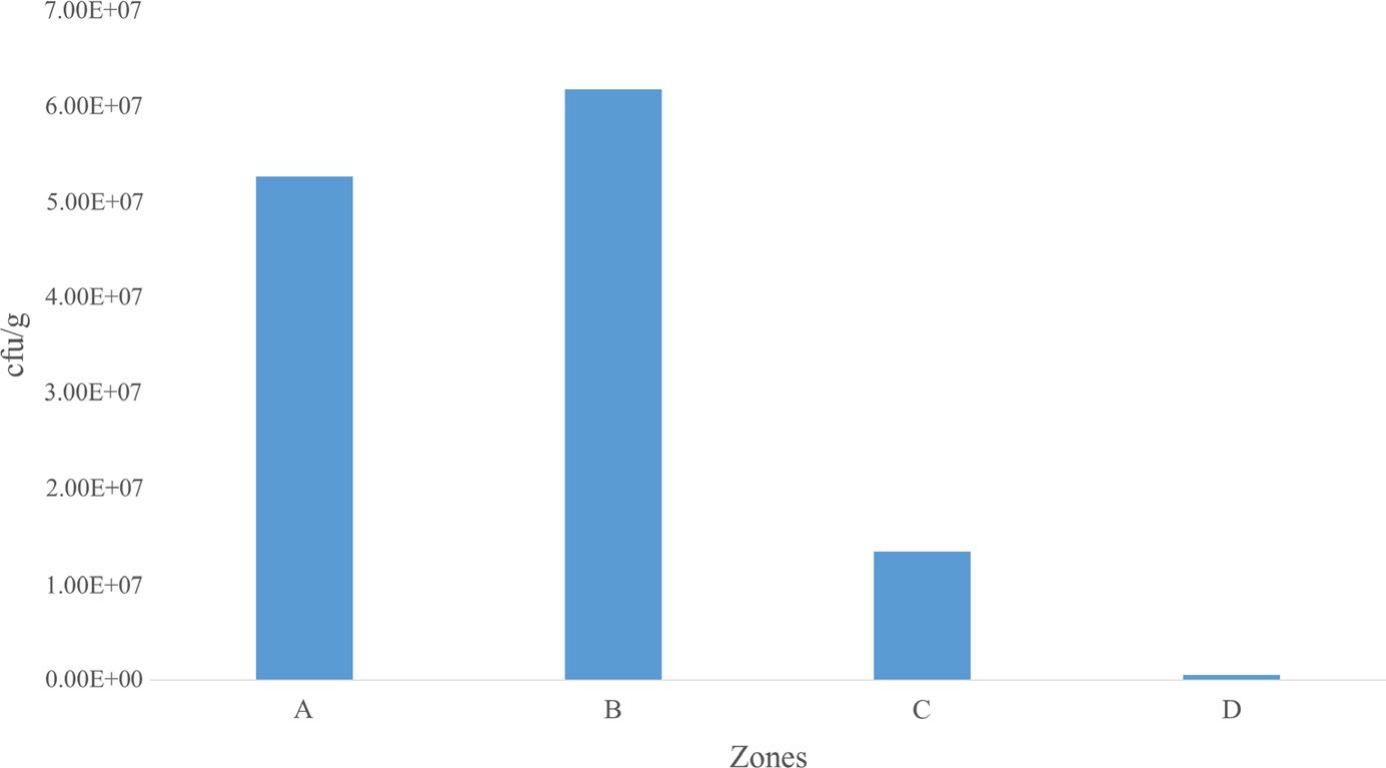
Mean counts of microbial load in T.Z recorded for the four zones. Cfu/g; colony‐forming units per gram. E; exponent

Specifically, the given zones demarcated in this study including A, B, C, and D recorded mean counts of 5.26 × 10^7^ ± 1.10 × 10^7^ cfu/g, 6.18 × 10^7^ ± 2.16 × 10^7 ^cfu/g, 1.34 × 10^7^ ± 2.31 × 10^6 ^cfu/g, and 5.16 × 10^5 ^± 2.98 × 10^5 ^cfu/g, respectively. Figure 2 illustrates the mean counts of microbial load in T.Z samples collected.

Table 1 shows the results of bacterial isolates with their mean values (±SEM) in cfu/g. The minimum count recorded for each bacteria was < 100, while the maximum count ranged from 2.3 × 10^6 ^to 1.25 × 10^6^cfu/g. Among the bacteria, *E. coli* count from the T.Z samples ranged from < 100 to 2.3 × 10^6^cfu/g with a mean (±SEM) of 4.49 × 10^5^± 5.72 × 10^4 ^cfu/g. *Shigella* spp count from the T.Z samples ranged from < 100 to 7.3 × 10^6 ^cfu/ml with a mean (±SEM) of 5.1 × 10^5^ ± 1.36 × 10^5^ cfu/g (Table 1). Again, the study showed that *Salmonella* spp count ranged from < 100 to 1.2 × 10^6 ^with a mean (±SEM) of 2.65 × 10^5 ^± 4.12 × 10^4 ^cfu/g. The counts of *Staphylococcus aureus* ranged from < 100 to 1.25 × 10^6 ^with a mean (±SEM) of 3.73 × 10^4^ ± 2.1 × 10^4^cfu/g (Table 1).

**Figure 3:**
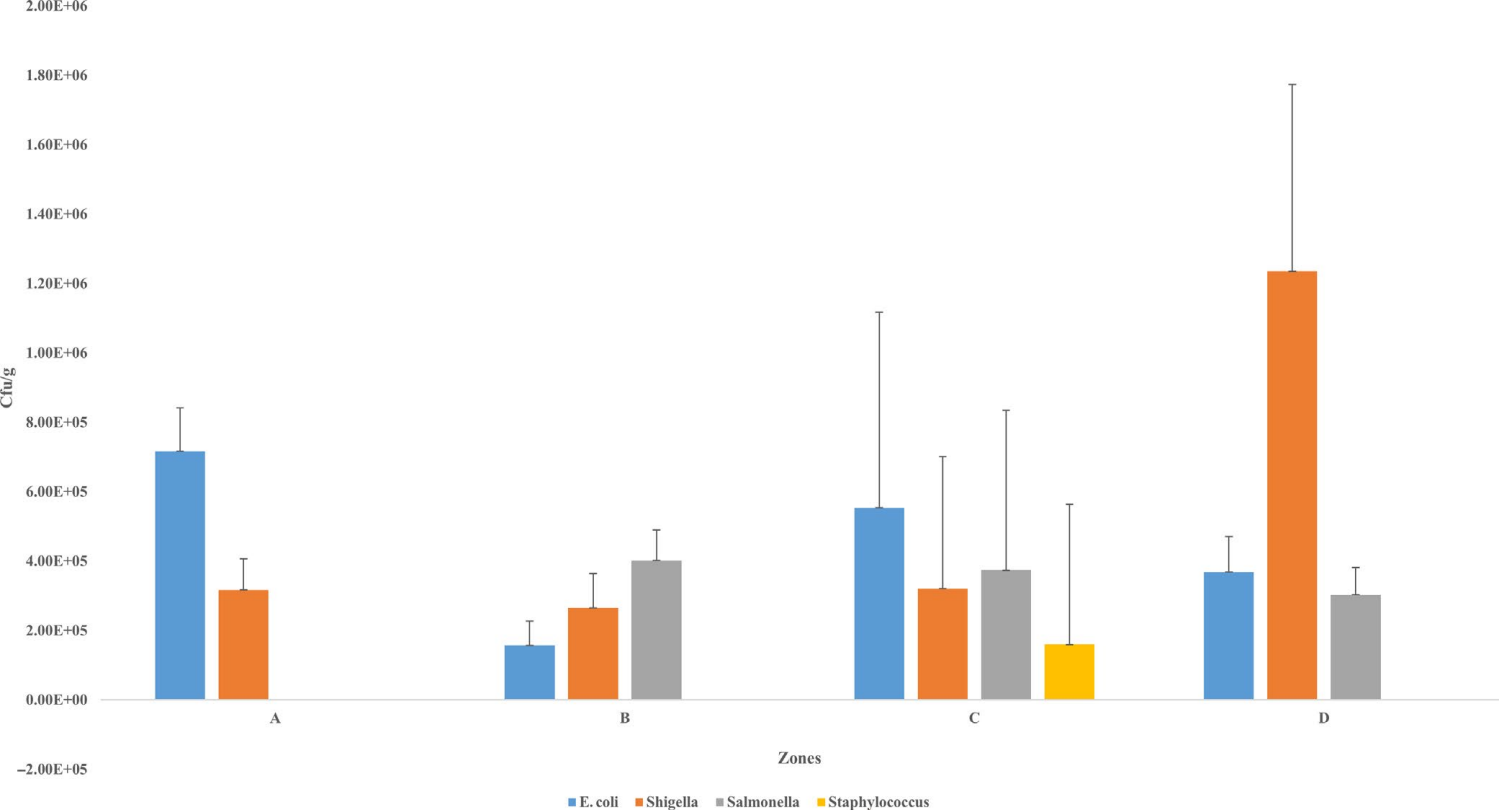
Mean counts of bacterial isolates across the four zones. cfu; colony‐forming units. E; exponent

Figure 3 illustrates the mean counts of bacterial isolates across the four zones which varied significantly (*p* = .001).

### 3.2 Occurrence of bacterial isolates in T.Z samples

Notably, *E. coli* was present in fifteen (15) samples of T.Z representing 50% of the total samples collected. Conversely, regarding the occurrence of *E. coli*, 15 samples (50%) indicated the absence of *E. coli* (Table 2). Significantly, *E. coli* recorded the highest occurrence when compared with the other bacteria considered in this study. *Shigella spp* occurred in eleven (11) samples representing 36.70% of the collected samples, while the remaining 19 (63.30%) samples recorded negative occurrence for *Shigella* spp (Table 2). Also, ten (10) samples representing 33.30% of the total samples recorded positive for *Salmonella,* while the remaining 20 samples representing 66.70% recorded negative (absent). Finally, for *Staphylococcus*, only one sample representing 3.33% recorded positive occurrence, while the remaining 29 samples thus 96.67% recorded negative occurrence, making *Staphylococcus aureus* the least prevalent bacteria among the samples collected (Table 2).

**Table 2:** Occurrence of bacterial isolates in T.Z samples

Bacteria	No. of samples (+)	Percentage (%)	No. of samples (−)	Percentage (%)
*Escherichia coli*	15	50	15	50
*Shigella* spp	11	36.7	19	63.3
*Salmonella* spp	10	33.3	20	66.7
*Staphylococcus aureus*	1	3.33	29	96.7

Note

Positive occurrence (+); Negative occurrence (−).

### 3.3 Antibiotic susceptibility of bacterial isolates

**Figure 4:**
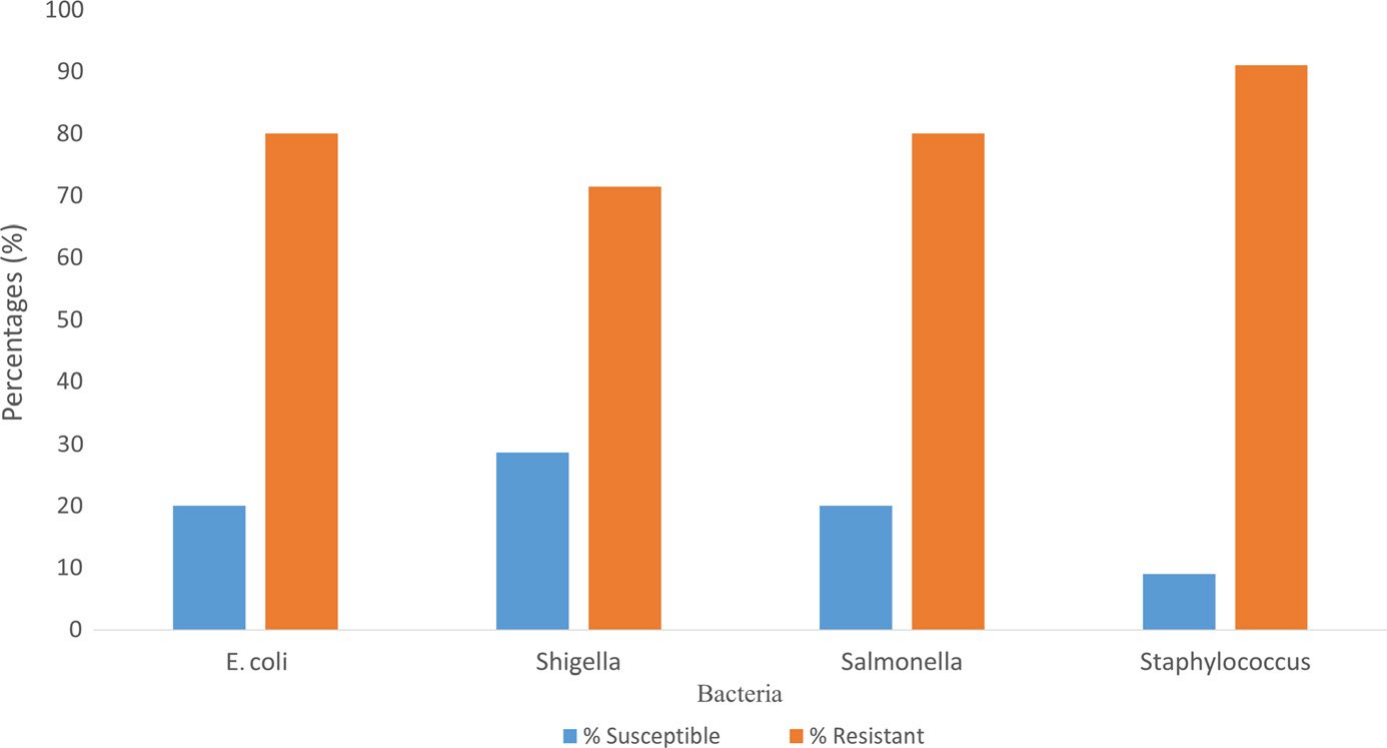
Percentages of the sensitivity of bacterial isolates to antibiotics. Not applicable

Figure 4 illustrates the susceptibility and resistivity of the bacterial isolates identified in the T.Z samples. *E. coli was* resistant to four (4) different antibiotics used out of five (5) representing 80%, and it was susceptible to only one (1) antibiotic (ciprofloxacin 5) representing 20%. Shigella was susceptible to two (2) antibiotics (ciprofloxacin 5 and tetracycline 30) out of seven (7) which represents 28.6%, and it was resistant to the remaining five (5) antibiotics also representing 71.4%. Also, Salmonella was resistant to four (4) antibiotics out of five (5) representing 80% and it was susceptible to one (1) antibiotic (ciprofloxacin 5) representing 20%. Again, Staphylococcus was susceptible to one (1) antibiotic (ciprofloxacin 5) out of eleven (11) which represents 9% and it was resistant to ten (10) antibiotics representing 91%.

## 4 DISCUSSION

The high levels of bacteria in the T.Z samples can be associated with the handling conditions of the food practiced by the food vendors. This was confirmed by personal observation upon several visits to the kitchens at the various vending sites. It was revealed that several people were involved at each stage in the preparation of the food just to hasten its readiness. Most of these people did not wash their hands in between different activities at the site including handling of money and using the washroom before handling the food. Also, the T.Z dumplings were made in nonsterile polythene bags and they were not stored properly. This conclusion was lean support from the findings of Chukuezi in 2010 and Oranusi et al in 2013 who indicated that food vendors are usually directly responsible for the contamination of food during the processing, preprocessing, and postprocessing, especially with the introduction of enteric bacteria. The characteristic of *E. coli* that allows it to change in response to different ecological conditions may be accountable for its presence in the T.Z analyzed. In a related study in Nigeria, Oluyege and Famurewa (2013) reported the presence of *E. coli* in 24.4% of cooked food samples from Ado‐Ekiti. Again, a study carried out in Uganda reported 60% of *E. coli* occurring in street‐vended foods (Mugampoza, Byarugaba, Nyonyintono, & Nakiho, 2013). In Ghana, street foods in Kumasi were reported to have been contaminated with 41.7% of *E. coli* (Boateng, 2014). These major findings raise a lot of concern for the safety of street foods consumed in most African countries, and this calls for a special attention from the relevant institutions and the general public.

With regard to the safety of T.Z for consumption, the Health Protection Agency in 2009 reported that for ready‐to‐eat foods placed on the market, *E. coli* counts ˃10^4 are unsatisfactory for consumption, counts ranging from 20 to 10^2^ are classified to be at the borderline, and counts ˂20 are described as satisfactory (Table 3). From this guideline, all the 50% (15) T.Z samples revealed the presence of *E. coli* were satisfactory. These findings in the present study differ from Temesgen, Haimanot, Derese, and Gebre (2016) who reported that 29.6% of samples contaminated with *E. coli* as satisfactory for consumption. The absence of *E. coli* in 15 samples in this study can be as a result of the observation of good hygienic practices by the vendors and the proper storage of T.Z.

**Table 3:** Standards for the interpretation of the levels of foodborne pathogens in vended foods (colony‐forming unit (cfu)/ml)

Criterion	Result (colony‐forming unit (cfu)/g or (cfu)/ml	
	Satisfactory	Unsatisfactory
*E. coli*	<20	>10^2^
*Shigella* spp	Not detected in 25g	Detected in 25g
*Salmonella* spp	Not detected in 25g	Detected in 25g
*Staphylococcus aureus*	<20	>10^4^

Note

Center for Food Safety, 2014.

Shigella is one of the four main pathogens that cause most moderate‐to‐severe diarrhea in Africa (Kotloff, Nataro, & Blackwelder, 2013). This study has detected *Shigella* spp in the T.Z samples collected from the central business district of Tamale. In the Silchar city of India, 19.64% of *Shigella* spp were isolated from ready‐to‐eat vended foods (Sharma & Mazumdar, 2014). According to the Center for Food Safety (2014), ready‐to‐eat foods that contain *Shigella* spp are attributable to cross‐contamination by food handlers and the contamination of the raw materials used to prepare the food. Also, ready‐to‐eat foods are wholesome for consumption if there is no *Shigella* spp isolated in 25g of the food sample, and if it is isolated, the food is unsatisfactory for consumption (Centre for Food Safety, 2014). From this guideline, all 36.7% (11) of T.Z samples indicated that the presence of Shigella spp was unsatisfactory (Table 3). The absence of *Shigella* spp in 19 of the T.Z samples in this study can be linked to proper hygienic practices by the vendors as well as the proper handling and storage of the food at those food vending establishments. The presence of *Salmonella* spp in food indicates a likely high health risk to consumers with regard to foodborne illnesses (CDC, 2016). This study has detected *Shigella* spp in the T.Z samples collected from the central business district of Tamale. In the Jigjiga city of eastern Ethiopia, it was reported that street‐vended foods contained 19.7% of *Salmonella* spp (Tesfaye, Emerie, Reta, & Asfaw, 2014).

According to the Center for Food Safety (2014), *Salmonella* spp detected in 25g of the sample is unsatisfactory and vice versa; therefore, all the 33.3% (10) samples of T.Z from which *Salmonella* spp was isolated were unsatisfactory (Table 3). The isolation of *Salmonella* spp in street food is likely to be as a result of inadequate processing and handling of raw materials and cross‐contamination (HPA, 2009) and poor hygienic measures on the part of food vendors. In contrast to the occurrence of *Salmonella* spp in this study, it was reported in a study conducted by Mutsuddy in 2016 that *Salmonella* spp were not isolated from all the street food samples examined.

One of the most common and frequent foodborne diseases in the world is the Staphylococcal foodborne disease which is a result of the ingestion of toxins produced by *Staphylococcus aureus* in foods (Jhalka, Tara, & Dipendra, 2014). The presence of *Staphylococcus aureus* in food poses potential health hazard to consumers. In Florida, it was recently reported that there was an outbreak of Staphylococcal foodborne disease at a science Olympiad (Linda, 2017). In the democratic republic of Congo, it was reported that food sold by street vendors in Kisangani was contaminated with 50% of *Staphylococcus aureus* (Makelele et al., 2015). This study has revealed the presence of *Staphylococcus aureus* in T.Z samples collected from the central business district of Tamale. These findings are critical and demand a drastic intervention from the relevant authorities.

In contrast to the results of this study, Abdullah, Khosiya, Nurainee, Zubaidah, and Amporn (2017) reported that cooked foods in southern Thailand were not contaminated with *Staphylococcus aureus*. According to the Center for Food Safety (2014), *Staphylococcus aureus* counts ˂20 are satisfactory (Table 3). In lieu of this guideline, the 3.33% T.Z sample from which *Staphylococcus aureus* was isolated is satisfactory. The presence of *Staphylococcus aureus* indicates a strong evidence of poor food handling and temperature control (HPA, 2009).

### 4.1 Antibiotic susceptibility of bacterial isolates

Given the inhibition zone results, the larger inhibition zones indicated lethality of the antibiotic disk and the smaller inhibition zones indicated the ineffectiveness of the antibiotic disk. All the tested bacteria identified in this study were 100% susceptible to ciprofloxacin which is in line with the report of Temesgen et al. (2016) where all isolates were also 100% sensitive to ciprofloxacin. 100% of the isolates were resistant to gentamycin which again contradicts the results of Temesgen et al. (2016) which reported 100% of the isolates being susceptible to gentamycin. The fact that bacterial isolates were susceptible to some antibiotics is an indication that the antibiotics remain the drug of choice for the management of most foodborne diseases (Temesgen et al., 2016). *E. coli* showed resistance to gentamycin, erythromycin, and other common antibiotics which is an indication that the treatment of *E. coli*‐related foodborne disease would be difficult in the central business district of Tamale. Also, *Staphylococcus aureus* showed resistance to ampicillin, penicillin, and chloramphenicol which is also an indication that the treatment of Staphylococcal‐borne disease or infections would be difficult in the central business district of Tamale. According to Barth, Melvin, James, and Mary (2009), a susceptible result shows that a bacterial isolate should respond to treatment with the antibiotic; it has shown susceptibility using the normally recommended dosage, and the resistant result indicates that the treatment of a bacterial infection with an antibiotic to which the isolate is resistant to in a normal dosage will not be achieved; hence, either the dosage is increased or the antibiotic is changed. From this study, 81% of all the isolates showed resistance to the antibiotics used and 19% showed susceptibility.

## 5 CONCLUSION

This study determined the microbial quality and antibiotic sensitivity of bacterial isolates in “Tuo‐Zaafi” (T.Z) vended in the central business district of Tamale. The results of the microbiological examination indicated differences in the level of contamination of the "Tuo‐Zaafi" samples.

There was a generally high resistance of the isolates to antibiotics presenting a major threat to public health. Also, the lack of proper food hygiene and safety training practiced by the T.Z vendors as well as the unhygienic surroundings of most vending sites can be possible reasons for higher levels of contamination observed. This study revealed that T.Z vended in the central business district of Tamale might pose a likely health risk to the consumers in terms of its microbiological quality. The contamination of the T.Z samples may be associated with unhygienic processing, preparation, and handling of the food, as well as its storage. Therefore, there is the need for the improvement of the standards of food hygiene and quality of vended T.Z in the central business district of Tamale.

## CONFLICT OF INTEREST

There were no competing interests among the authors.

## AUTHORS’ CONTRIBUTIONS

The lead author (AR) carried out the laboratory analysis. She reviewed and provided the relevant literature for the study. The second author (CSJ) supervised the study from its conception to the end, and he was also responsible for all the necessary corrections in the write‐up. The third author (AG) assisted in carrying out the experiment, organizing the write‐up, and making the needed corrections, as well as submitting it to this journal for publication.

## ETHICAL APPROVAL

Ethics approval is not applicable, and no human or animal subjects were used in this study. The study's protocols were ethically reviewed by Ghana Standards Board and Food and Drugs Authority.

## CONSENT FOR PUBLICATION

Not applicable.

## Data Availability

Datasets obtained and analyzed in this study are available within the main text, and any other information would be provided on request.

## References

[fsn31216-bib-0001] Abakari, G. , Cobbina, S. J. , & Yeleliere, E. (2018). Microbial quality of ready‐to‐eat vegetable salads vended in the central business district of Tamale, Ghana. International Journal of Food Contamination, 5, 3 10.1186/s40550-018-0065-2

[fsn31216-bib-0002] Abdullah, D. D. , Khosiya, S. , Nurainee, H. , Zubaidah, H. , & Amporn, T. (2017). Microbiological quality of cooked foods and drinks sold in higher educational institutions around Yale, Pattani and Narathiwat provinces. Southern Thailand., 1(1868).

[fsn31216-bib-0003] Adetunji, V. O. , Hezekiah, K. A. , Charity, A. A. , & Tajudeen, O. I. (2014). Bacterial load and antimicrobial profile of *Escherichia coli* and *Listeria* spp isolates from muscle tissues of slaughtered cattle at a major abattoir in Ibadan, South‐Western Nigeria. Journal of Basic and Applied Sciences, 10, 1927–5129.

[fsn31216-bib-0004] Al‐Waili, N. , Al‐Ghamdi, A. , Ansari, M. J. , Al‐Attal, Y. , & Salom, K. Y. (2012). Synergistic effects of honey and propolis toward drug multi‐resistant Staphylococcus aureus, *Escherichia coli* and Candida albicans isolates in single and polymicrobial cultures. International Journal of Medical Sciences, 9(9), 793–800. 23136543 10.7150/ijms.4722 PMC3491439

[fsn31216-bib-0005] APHA (American Public Health Association) (2008) Standard methods for the examination of water and wastewater (19th edn). Washington, DC: American Public Health Association (APHA). Microbiological Quality of selected street vended foods in Port Harcourt metropolis, Rivers State, Nigeria C.

[fsn31216-bib-0006] Barth, L. R. , Melvin, W. , James, H. J. , & Mary, J. F. (2009). Antimicrobial susceptibility testing: A review of general principles and contemporary practices. Oxford Journals, 49(11), 1749–1755. 10.1086/647952 19857164

[fsn31216-bib-0007] Bereda, T. W. , Emerie, Y. M. , Reta, M. A. , & Asfaw, H. S. (2014). Microbiological safety of street vended foods in Jigjiga city, Eastern Ethiopia. Ethiopian Journal of Health Sciences, 26(2), 161–170. 10.4314/ejhs.v26i2.10 PMC4864345 27222629

[fsn31216-bib-0008] Boateng, A. E. (2014). Assessment of food hygiene practices by street food vendors and microbial quality of selected foods sold. B.A nursing and psychology. Thesis. Kwame Nkrumah University of Science and Technology. Ghana. 64.

[fsn31216-bib-0009] Center for Disease Control (CDC) (2016). Control and prevention of diseases. [accessed 6th June, 2018].

[fsn31216-bib-0010] Center for Science in the Public Interest (CSPI) (2013). Antibiotic resistance in foodborne outbreaks, Washington DC. Retrieved from: https://cspi;net.org/resource/antibiotic-resitance. [accessed 15th March, 2018].

[fsn31216-bib-0011] Centers for Disease Control and Prevention (CDC) (2000). National center for health statistics, division of health examination statistics. Retrieved from http://www.cdc.gov/nchs/products/pubs/pubd/hus/tables/2000/updated/00hus069.pdf.

[fsn31216-bib-0012] Center for Food Safety . (2014). Microbiological guidelines for food; for ready-to-eat food in general and specific food items. Food and Environmental Hygiene Department, pp 8–12. https://www.cfs.gov.hk/english/food_leg/files/food_leg_Microbiological_Guidelines_for_Food. [accessed on 25 January 2018].

[fsn31216-bib-0013] Chapentier, E. , & Courvalin, P. (1999). Antibiotic resistance as a biological hazard. European Food Safety Authority Journal, 43(9), 2103–2108.

[fsn31216-bib-0014] Chukuezi, C. O. (2010). Food safety and hygienic practices of street food vendors in Owerri, Nigeria. Studies in Sociology and Science, 1(1), 50–57.

[fsn31216-bib-0015] FAO/WHO (2010). Framework for developing national food safety emergency response plans. Joint FAO/WHO publication. ISBN 9789‐241‐500‐357.

[fsn31216-bib-0016] Ghana statistical service (2010). 2010 census report. Retrieved from: www.statsghana.gov.gh [accessed 14 November 2017].

[fsn31216-bib-0017] Giovannucci, D. , & Satin, M. (2000). Food Quality Issues : Understanding HACCP and Other Quality Management Techniques. A Guide to Developing Agricultural Markets and Agro-Enterprises. Washington, DC: World Bank © World Bank. https://openknowledge.worldbank.org/handle/10986/17702. License: CC BY 3.0 IGO.

[fsn31216-bib-0018] Health Protection Agency (2009). Guidelines for assessing the microbiological safety of ready‐to‐eat foods placed on the market. [accessed 6 June 2018].

[fsn31216-bib-0019] Jhalka, K. , Tara, C. S. , & Dipendra, T. (2014). Staphylococcus and Staphylococcal foodborne disease: An increasing challenge in public health. Biomedical research international (review article). 9p. Available from: 2018, doi: 10.1155/2014/827‐965. [accessed 22 June].

[fsn31216-bib-0020] Kotloff, K. L. , Nataro, J. P. , & Blackwelder, W. C. (2013). Burden and aetiology of Diarrhoeal disease in infants and young children in developing countries (the Global Enteric Multicenter Study, GEMS): A prospective, case‐control study. The Lancet, 382(9888), 209–222. 10.1016/S0140-6736(13)60844-2 23680352

[fsn31216-bib-0021] Liinaghanas (2014). Northern Ghanaian's most common dish T.Z with vegetable soup. Retrieved from: https://foodforthoughtmondo.wordpress.com/2014/11/17/northern-ghanaian-most-common-dish-tz-with-vegetable-soup/. [accessed 13 November 2017].

[fsn31216-bib-0022] Linda, L. (2017). Staphylococcus aureus sickened students at science olympiad. Available from, 9(5), 285‐290. https://foodpoisoningbulletin.com.

[fsn31216-bib-0023] Makelele, L. K. , Kazadi, Z. A. , Oleko, R. F. , Rossette, K. A. M. , Koto‐te‐Nyiwa, N. , & Bongo, N. G. (2015). Microbiological quality of food sold by street vendors in Kisangani, DR Congo. African Journal of Food Science, 10(2), 285-290.

[fsn31216-bib-0024] Mugampoza, D. , Byarugaba, G. W. B. , Nyonyintono, A. , & Nakiho, P. (2013). Occurrence of *E. coli* and *Salmonella* spp in street vended foods and general hygienic and trading practices in Nakawa Division. Uganda. American Journal of Food and Nutrition, 3(3), 167-175.

[fsn31216-bib-0025] Mutsuddy, S. (2016). Isolation and Identification of enteric bacteria in different street vended foods collected from different private universities in Dhaka city, Bangladesh.

[fsn31216-bib-0026] Ogidi, O. C. , Victor, O. O. , & Bamidele, J. A. (2016). Microbial quality and antibiotic sensitivity pattern of isolated microorganisms from street foods sold in Akure Metropolis, Nigeria. Jordan Journal of Biological Sciences, 9(1995–6675), 227–234.

[fsn31216-bib-0027] Oluyege, O. A. , & Famurewa, O. (2013). Microbial contamination and antibiotic resistance in enteric pathogens isolated from cooked foods sold in eateries in ado-ekiti, Nigeria. British Microbiology Research Journal, 6(4), 236–246. 10.9734/BMRJ/2015/7351

[fsn31216-bib-0028] Oranusi, S. U. , Oguoma, O. I. , & Agusi, E. (2013). Microbiological quality assessment of foods sold in student's cafeterias. Global Research Journal of Microbiology, 3(1), 1–7.

[fsn31216-bib-0029] Sharma, J. , & Mazumdar, J. A. (2014). Assessment of bacteriological quality of ready to eat food vended in streets of Silchar city, Assam, India. India Journal of Medical Microbiology, 32(2), 169–171. 10.4103/0255-0857.129809 24713905

[fsn31216-bib-0030] Temesgen, E. , Haimanot, T. , Derese, D. , & Gebre, K. (2016). Bacteriological quality of street foods and Antimicrobial resistance of isolates in Hawassa Ethiopia . Ethiopian Journal of Health Sciences, 26(6), 533–542. 10.4314/ejhs.v26i6.5 28450768 PMC5389072

[fsn31216-bib-0031] WHO . (2010). Framework for developing national food safety emergency response plans. Joint FAO/WHO publication. ISBN 9789-24 1-500-35.

[fsn31216-bib-0032] WHO (2011). World health statistics report. [accessed 18 May 2018].

[fsn31216-bib-0033] WHO (2015). World health day: Food safety! What you should know. Retrieved from https://www.who.int>campaigns>2015. [accessed 25 January 2018].

[fsn31216-bib-0034] WHO (2017). Food safety (reviewed in October 2017). [accessed 16 March 2018].

[fsn31216-bib-0035] WHO (2018). Antibiotics resistance. News Room. Retrieved from https://www.who.int/news-room/fact-sheets/detail/antibiotic-resistance [accessed 12 July 2019].

